# Potential of *Asparagopsis armata* as a Biopesticide for Weed Control under an Invasive Seaweed Circular-Economy Framework

**DOI:** 10.3390/biology10121321

**Published:** 2021-12-13

**Authors:** Bernardo Duarte, João Carreiras, Eduardo Feijão, Ricardo Cruz de Carvalho, Ana Rita Matos, Vanessa F. Fonseca, Sara C. Novais, Marco F. L. Lemos

**Affiliations:** 1 MARE—Marine and Environmental Sciences Centre, Faculty of Sciences of the University of Lisbon, Campo Grande, 1749-016 Lisbon, Portugal; jgcarreiras@fc.ul.pt (J.C.); emfeijao@fc.ul.pt (E.F.); rfcruz@fc.ul.pt (R.C.d.C.); armatos@fc.ul.pt (A.R.M.); vffonseca@fc.ul.pt (V.F.F.); 2 Departamento de Biologia Vegetal, Faculdade de Ciências da Universidade de Lisboa, Campo Grande, 1749-016 Lisbon, Portugal; 3 BioISI—Biosystems and Integrative Sciences Institute, Plant Functional Genomics Group, Departamento de Biologia Vegetal, Faculdade de Ciências da Universidade de Lisboa, Campo Grande, 1749-016 Lisboa, Portugal; 4 Departamento de Biologia Animal, Faculdade de Ciências da Universidade de Lisboa, Campo Grande, 1749-016 Lisbon, Portugal; 5 MARE—Marine and Environmental Sciences Centre, ESTM, Polytechnic of Leiria, 2520-641 Peniche, Portugal; sara.novais@ipleiria.pt

**Keywords:** biorefinery, herbicide, marine biotechnology, pesticide, oxidative stress

## Abstract

**Simple Summary:**

The invasive seaweed *Asparagopsis armata* has the potential to be used as a biopesticide. The application of its exudate shows severe impacts on energetic and carotenoid metabolism and induces significant oxidative stress in a model weed. This points to the potential use of this macroalga as a resource for a biopesticide cocktail, for sustainable and eco-friendly weed control and as a substitute for the chemical pesticides widely used nowadays.

**Abstract:**

Marine macroalgae have been increasingly targeted as a source of bioactive compounds to be used in several areas, such as biopesticides. When harvesting invasive species, such as *Asparagopsis armata*, for this purpose, there is a two-folded opportunity: acquiring these biomolecules from a low-cost resource and controlling its spreading and impacts. The secondary metabolites in this seaweed’s exudate have been shown to significantly impact the physiology of species in the ecosystems where it invades, indicating a possible biocidal potential. Considering this in the present work, an *A. armata* exudate cocktail was applied in the model weed *Thellungiella halophila* to evaluate its physiological impact and mode of action, addressing its potential use as a natural biocide. *A. armata* greatly affected the test plants’ physiology, namely, their photochemical energy transduction pathway (impairing light-harvesting and chemical energy production throughout the chloroplast electron transport chain), carotenoid metabolism and oxidative stress. These mechanisms of action are similar to the ones triggered when using the common chemical pesticides, highlighting the potential of the *A. armata* exudate cocktail as an eco-friendly biopesticide.

## 1. Introduction

Due to the demand for higher agricultural productivity to supply a growing population worldwide, pesticides and agrochemicals, in general, became a paramount component of global agriculture systems over the last century [1]. Simultaneously, in the last decades, agrochemical residues spread in the environment, causing the contamination of terrestrial and aquatic ecosystems [2], with significant effects on wildlife in all the ecosystem compartments [3,4]. Pesticides typically exhibit a mechanism for toxic action that is not restricted to target species or pests, with toxic effects exerted also on nontarget organisms, causing the impoverishment of biodiversity and ecosystems health [1]. Moreover, pesticides tend to be environmentally persistent, remaining for long periods in soils and/or sediments, and eventually leaching to aquatic environments, leading to their accumulation throughout the trophic webs, with devastating toxic effects at the population level [3]. Nowadays, the search for a more eco-friendly solution has been the goal of many researchers worldwide, with biopesticides gaining an increased interest for developing environmentally friendly, safe and Integrated Crop Management (ICM)-compatible approaches and tactics for pest management [5]. Several aspects highlight biopesticides as safe alternatives to classical synthetic solutions, with lower environmental risk at the top of the list of advantages [6]. These bio-solutions can come from a wide variety of organisms, with several products already being released and registered in the agromarket [6]. The natural origin of these products has also boosted the public acceptance of biopesticides, promoting their increased use worldwide [7].

The marine realm has been targeted for the discovery of new and differentiated biomolecules, with several applications ranging from the pharmaceutical and cosmetic to the agricultural sectors [8]. In particular, marine algae have been targeted as an excellent natural source of biomolecules in different aspects of agricultural areas [9]. Among these applications, marine macroalgae have been targeted because they produce a large array of biologically active biocidal substances [9], opening the door for the search of biopesticide cocktails from marine macroalgae origin.

The red seaweed *Asparagopsis armata* (Harvey, 1855) has been found in the European coasts since 1925 and can also be found in the Northeast Atlantic (from the Shetland Islands to Morocco), Mediterranean, South Africa, Middle East, and Indo-Pacific region, far away from its original endemic habitat on Western Australia and New Zealand coastlines [10]. Additionally, due to its highly invasive character and high biomass availability, it has the potential to displace key species from their natural habitat [11,12], and severe toxic effects of its exudates have been shown for marine invertebrates, triggering inflammation and immunity responses [13] and negatively impacting their energetic metabolism [14]. These toxic compounds are synthesized and stored in vacuoles within gland cells [15] including various compounds, mainly bromoform and smaller quantities of other bromine-, iodine-, and chlorine-containing methane, ethanol, ethane, acetones, acetaldehydes, 2-acetoxypropanes, acroleins, propenes, epoxypropanes, and butenones [15,16]. These secondary metabolites are known for their antimicrobial and cytotoxic properties, among others [10], which further points to the possible plant biocidal potential of this mixture. Due to their negative impact on coastal ecosystems worldwide, there is a growing demand for the development of efficient control strategies to regulate invasive macroalgae [10,17]. *Asparagopsis armata* produces a myriad of secondary metabolites with proven biotechnological potential, being already a resource with high market demand due to its cosmeceutical potential and trending anti-methanogenic activity in ruminants (biotechnological potential and uses extensively reviewed in [18]). Still, current knowledge on the biotechnological use of its released exudate is lacking, which could represent a two-folded opportunity: to use its exudate and recover its by-products (the whole biomass) for other biotechnological uses—current and new—following a smart biorefinery concept and the much requested circular economy approach, thus specifically contributing to the blue growth strategy [19] and a myriad of sustainable development goals. Having in mind their potential application as a source of bioactive compounds, allied to the need to control invasive macroalgae populations, the valuation of these natural resources for industrial and agricultural applications could be a suitable strategy to reduce their abundance, obtaining both environmental and economic benefits [10,17].

Considering the abovementioned facts, the present work aims to test the potential of an *A. armata* exudate cocktail as a biopesticide for weed control, evaluating its effects on the physiology of a model plant, *Thellungiella halophila*, which is a close relative to *Arabidopsis thaliana* (the model glycophyte weed) and has been ascribed as a new model marine/halophyte plant due to its tolerance to saline environments; therefore, it is the ideal plant to assess the exudates produced in seawater, reducing the potential artefacts imposed by potential salt stress [20].

## 2. Materials and Methods

### 2.1. Asparagospsis armata Collection and Preparation of Exudates

*Asparagopsis armata* gametophytes were collected by scuba diving from the protected marine area around the Berlenga Island, Peniche, Portugal (39°25′03.0″ N, 9°30′23.6″ W). In the lab, after being cleaned and sorted, four aquaria with 5 kg of *A. armata* and 50 L of artificial seawater (distilled water with Premium REEF salt (TMC, Lisbon, Portugal) adjusted to 35 PSU) were left in the dark at 20 °C. After 12 h, the seaweed was removed, and the water from the different aquaria was pooled and sieved for bigger particles, followed by filtration through a 0.45 µm cellulose acetate membrane filter (Whatman, Maidstone, UK). The exudate was then kept in PET bottles at −20 °C until further use and represented the 100% stock concentration.

### 2.2. Thellungiella halophila Culture and Exposure

To evaluate the effects of the *A. armata* exudate, the plant species *T. halophila* was selected due to its high degree of tolerance to salt stress (halophyte species), preventing any masking effects from the saltwater exposure (exudate carrier solution) [21]. Plants of *T. halophila* were grown under controlled conditions: 22/20 °C, 10 h/14 h day/night, 250 µmol s^−1^ m^−2^ light energy supplied by OSRAM-HQIE lamps, 60% relative air humidity. Seeds were sown directly in pots containing a mixture of soil and perlite. Three seedlings, as morphologically identical as possible, were left to grow in each pot. They were watered daily with a nutritive solution (N:P:K4:5:7, Bo 0.01%, Cu 0.002%, Fe 0.02%, Mn 0.01%, Mo 0.001%, Zn 0.002%). The plants were left to grow under these conditions and exposure occurred only after the complete development of the 4 main rosette leaves (fully expanded). Exposure occurred for 3 days by spraying each plant (N = 5 per treatment) with 2 mL of exudate at each of the target concentrations every day. The final concentrations were attained by diluting the stock solution exudate (produced as detailed in Section 2.1 and considered as 100% concentration) with the artificial water used in Section 2.1. A control concentration of 0% (artificial seawater, only to evaluate any potential effect from the exudate carrier) and exudate concentrations of 1%, 2% and 3% were prepared freshly from frozen concentrate exudate stock aliquots. All samples for the biochemical analysis were collected at the end of the experiment and immediately flash-frozen in liquid nitrogen and stored at −80 °C until further analysis.

### 2.3. Plant Primary Photochemistry

At the end of the experimental period, Pulse Amplitude Modulated (PAM) midday chlorophyll fluorescence measurements were performed using a FluorPen FP100 (Photo System Instruments, Drásov, Czech Republic), on 30 min dark-adapted attached leaves. The polyphasic rise in fluorescence (OJIP) transient in dark-adapted leaves was attained using the pre-programmed OJIP protocol of the FluorPen [22]. The OJIP transient (or Kautsky curves) depicts the rate of reduction kinetics of various PSII components. This is obtained when a dark-adapted leaf is illuminated with the saturating light intensity of 3500 μmol m^−2^ s^−1^, after which it exhibits a polyphasic rise in fluorescence (OJIP): level O represents all the open reaction centres (RCs) at the onset of illumination with no quinone A (Q_A_) reduction (fluorescence intensity lasts for 10 ms); O to J transient indicates the net photochemical reduction of Q_A_ (the stable primary electron acceptor of PS II) to Q_A_^−^ (lasts for 2 ms); the J to I transition is due to all the reduced states of the closed RCs, such as Q_A_^−^ Q_B_^−^, Q_A_ Q_B_^2−^ and Q_A_^−^ Q_B_ H_2_ (lasts for 2–30 ms); and the P-step coincides with the maximum concentration of Q_A_^−^ Q_B_^2^ with the plastoquinol pool maximally reduced, also reflecting the balance between the light incident at the PS II side and the rate of utilization of the chemical (potential) energy and the rate of heat dissipation [23]. From this analysis, several variables can be attained using FluorPen 1.1 software (Table 1).

### 2.4. Leaf Pigment Profile

Freeze-dried and ground leaf samples were extracted with 100% acetone and subjected to an ultra-sound bath (VWR International, Germany) for 1 min to ensure complete disaggregation of the leaf material. Extraction occurred in the dark for 24 h at −20 °C, after which the samples were centrifuged at 4000× *g* at 4 °C for 15 min. Supernatants were scanned from 350 nm to 750 nm in 1 nm steps, using a dual-beam spectrophotometer (Shimadzu UV/VIS UV1601 Spectrophotometer, Country) and the absorbance data were analysed employing the Gauss-Peak Spectra (GPS) method [22,24]. The De-Epoxidation State (DES) was calculated as

DES = (Antheraxanthin + Zeaxanthin)/(Violaxanthin + Antheraxanthin + Zeaxanthin)

### 2.5. Leaf Fatty Acid Profile

Leaf fatty acid analyses were performed by direct trans-esterification of the leaf samples, as previously described [22,25,26]. Fatty acid methyl esters (FAMEs) were prepared in glass tubes containing the internal standard heptadecanoate (C17:0), methanol, and sulphuric acid (97.5:2.5, *v/v*), at 70 °C for one hour. After cooling down, the FAMEs were extracted by adding petroleum ether (boiling point 80–100 °C, Sigma) and Milli-Q water (18.2 MΩ cm), vortexed, and centrifuged at 4000× *g* for 5 min. The upper layer was dried under a nitrogen stream in a water bath set to 37 °C. After evaporation, 50 µL of hexane was added to the residue and 1 µL of the solution separated in a gas chromatograph (Varian 430-GC, Palo Alto, CA, USA) equipped with a hydrogen flame-ionization detector using a fused silica 0.25 mm i.d. × 50 m capillary column (WCOT Fused Silica, CP-Sil 88 for FAME; Varian). The double-bound index (DBI) was calculated using the equation:

$$\mathrm{DBI}=\frac{2\times \left(\left(16:1t+18:1\right)+2\times 18:2+3\times \left(18:3+16:3\right)\right)}{100}$$

### 2.6. Leaf Oxidative Stress Biomarkers

Enzyme extractions were performed according to Tiryakioglu et al. [27], at 4 °C. Frozen leaves were homogenized in 50 mM sodium phosphate buffer (pH 7.6) supplemented with 0.1 mM Na-EDTA in a ceramic mortar with a proportion of 500 mg (FW) to 8 mL respectively. The homogenate was centrifuged at 8890× *g* for 10 min at 4 °C, and the supernatant was transferred to a test tube and used for the antioxidant enzyme analyses.

The enzyme activity measurements of catalase (CAT, EC.1.11.1.6.), ascorbate peroxidase (APx, E.C. 1.11.1.11), guaiacol peroxidase (GPX, E.C. 1.11.1.7), glutathione reductase (GR, E.C. 1.8.1.7) and superoxide dismutase (SOD, E.C. 1.15.1.1) were performed in a microplate reader spectrophotometer (Epoch 2 Microplate Reader, BioTek Instruments, VT, USA). Catalase activity assays were performed according to the method of Teranishi et al. [28], by monitoring the H_2_O_2_ consumption and consequent decrease in absorbance at 240 nm (molar extinction coefficient of 39.4 mM^−1^ cm^−1^). Ascorbate peroxidase was measured according to Tiryakioglu et al. [27], by observing the ascorbate oxidation and consequent absorbance reduction at 290 nm (molar extinction coefficient of 2.8 mM^−1^ cm^−1^). Guaiacol peroxidase measurement was performed according to Bergmeyer et al. [29], by monitoring the guaiacol oxidation products formation and its increase in absorbance at 470 nm (molar extinction coefficient of 26.6 mM^−1^ cm^−1^). Superoxide dismutase total activity was assayed according to the method of Marklund and Marklund [30], by measuring the oxidation rate of pyrogallol and its increase in absorbance at 325 nm. The autoxidation of pyrogallol was read without an enzymatic extract during the same period and time interval for comparison enabling. Glutathione reductase activity was assayed by monitoring glutathione-dependent oxidation of NADPH and its decrease in absorbance at 340 nm in 0.2 mM NADPH, and 0.5 mM oxidized glutathione [31]. Protein quantification was determined using the Bradford method [32]. An oxidative ratio was calculated considering the intricate reactions occurring between SOD and the peroxidasic enzymes. In short, SOD dismutates the superoxide anion into hydrogen peroxide, which is subsequently quenched by peroxidases (CAT, APx and GPx). Thus, an oxidative ratio can reflect the balance between this hydrogen peroxide production and consumption and was calculated according to the following equation:

$$\mathrm{Oxidative}\;\mathrm{ratio}=\frac{\mathrm{SOD}}{\mathrm{CAT}\;+\;\mathrm{APx}\;+\;\mathrm{GPx}}$$

where, SOD, CAT, APx and GPx are the activity values determined for superoxide dismutase, catalase, ascorbate peroxidase and guaiacol peroxidase, respectively.

Lipid peroxidation quantification was performed in leaf samples by the thiobarbituric acid reactive substances (TBARS) method according to Heath and Packer [33]. First, leaf samples were homogenized in a freshly prepared thiobarbituric acid (TBA) solution (0.5% (*w/v*) TBA in 20% (*w/v*) trichloroacetic acid), in a proportion of 100 mg FW to 2 mL of solution. The homogenate was incubated for 30 min at 95 °C, cooled on ice to stop the reaction, and centrifuged at 4000× *g* for 5 min at 4 °C. The absorbance was read at 532 nm and 600 nm in a Shimadzu UV-1601 spectrophotometer (country). TBARS were expressed as malondialdehyde (MDA) equivalents and the concentration was calculated using the molar extinction coefficient, 155 mM^−1^ cm^−1^:

$$A_{532\;\mathrm{nm}}-\;A_{600\;\mathrm{nm}}=\left[\mathrm{MDA}\right]\mathrm{mM}\;\times \;\varepsilon \mathrm{MDA}$$

### 2.7. Statistical Data Analysis

Spearman correlation coefficients and statistical significance among the biophysical and biochemical traits of individuals were computed using the corrplot package [34] in R-Studio Version 1.4.1717. Boxplots with probability density of the data at different values smoothed by a kernel density estimator were computed and plotted using the ggplot2 package [35] in R-Studio Version 1.4.1717. Non-parametric Kruskal–Wallis with Bonferroni posthoc tests, for comparisons between the variables and samples exposed to different exudate concentrations, were performed in R-Studio Version 1.4.1717 using the agricolae package [36]. Canonical Analysis of Principal Coordinates (CAP) was used to evaluate the ability to successfully classify individuals according to the exudate exposure conditions using each of the considered biochemical and biophysical traits. This multivariate approach is insensitive to heterogeneous data and frequently used to compare different sample groups using the intrinsic characteristics (biochemical and biophysical traits) of each group [26,37,38]. Multivariate statistical analyses were conducted using Primer 6 software [39].

## 3. Results

### 3.1. Primary Photochemistry

**Figure 1:**
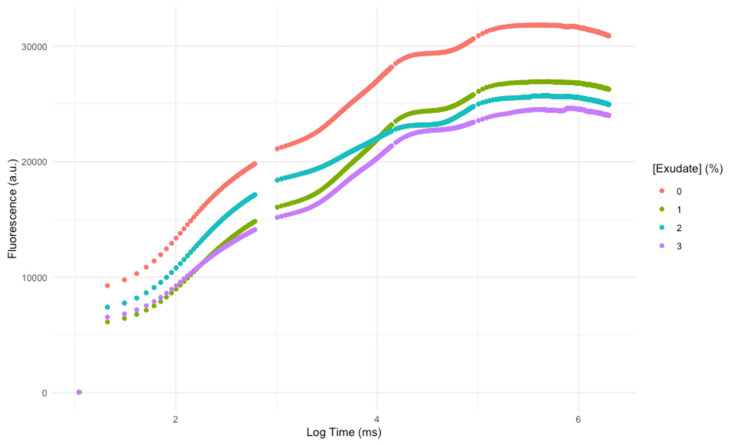
Kautsky chlorophyll *a* fluorescence induction curves of *Thellungiella halophila* dark-adapted leaves exposed to *Asparagopsis armata* exudate at the proposed target concentrations at the end of the trial period (average; N = 5 per treatment).

Observing the chlorophyll *a* fluorescence induction curve (Figure 1), it is evident that the overall photochemical profile of the leaves was affected by the *A. armata* spray application. Not only the values of fluorescence were lower, but also the shape of the curves and their inflexions were affected. This translates into changes in the key photochemical processes and variables.

**Figure 2:**
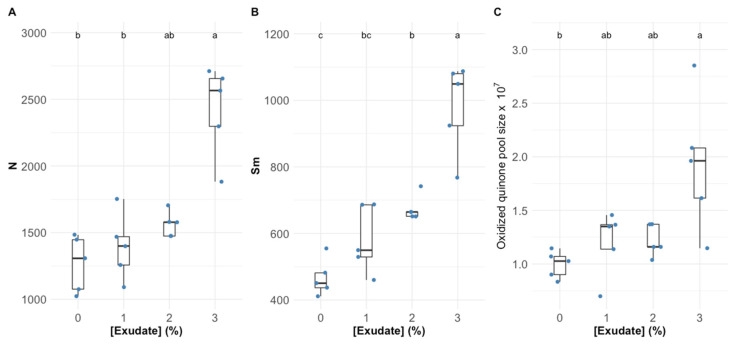
*Thellungiella halophila* dark-adapted leaves’ reaction centres turnover rate, N (**A**), the energy needed to close all the reaction centres, Sm (**B**), and the oxidized quinone pool size (**C**) exposed to *Asparagopsis armata* exudate at the proposed target concentrations at the end of the trial period (N = 5 per treatment; letters denote significant differences between treatments at *p* < 0.05).

Analysing the main features that modulate electron flow through the electron transport chain (ETC), it is possible to observe that the exudate application led to a significant increase in the reaction centre turnover rate (Figure 2A), the energy needed to close all reaction centres (Figure 2B), and oxidized quinone pool size (Figure 2C), this being more evident in the plants subjected to the higher concentration of exudate. 

**Figure 3:**
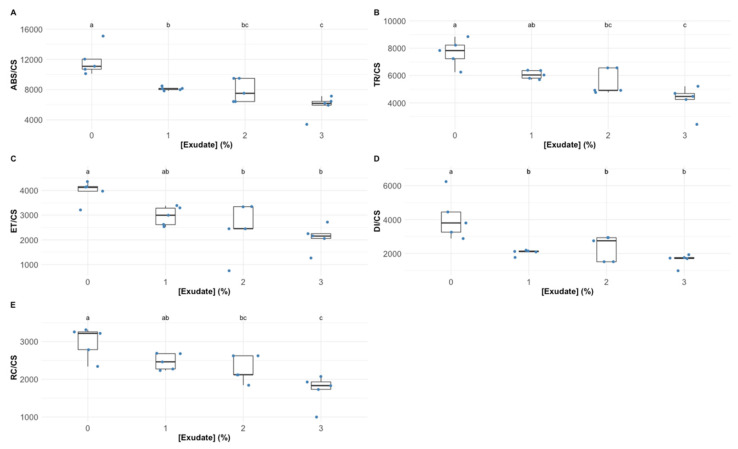
*Thellungiella halophila* phenomological energy fluxes absorbed (ABS/CS) (**A**), trapped (TR/CS) (**B**), transported (ET/CS) (**C**), and dissipated (DI/CS) (**D**), and the number of available reaction centres per cross-section (RC/CS) (**E**) in leaves exposed to *Asparagopsis armata* exudate at the proposed target concentrations at the end of the trial period (N = 5 per treatment; letters denote significant differences between treatments at *p* < 0.05).

Considering the phenomological energy fluxes that determine the plants’ primary photochemistry, a similar trend could be observed (Figure 3). The absorbed energy flux (ABS/CS) suffered a severe depletion even in the plants exposed to the lowest exudate concentration, this decrease being more marked in the plants exposed to the highest exudate concentration (Figure 3A). The trapped (TR/CS) and transported (ET/CS) energy fluxes also suffered a significant depletion, especially in the plants exposed to exudate concentrations above 1% (Figure 3B,C respectively). Regarding the dissipated energy flux (DI/CS; Figure 3D), it dropped significantly after the application of 1% exudate, without further significant decreases with the exudate increasing dose. The decrease in the energy fluxes was followed by a decrease in the number of oxidized reaction centres per leaf cross-section (RC/CS), which was more marked in the plants exposed to 2% and 3% exudate concentrations (Figure 3E).

### 3.2. Leaf Pigment Profile

**Figure 4:**
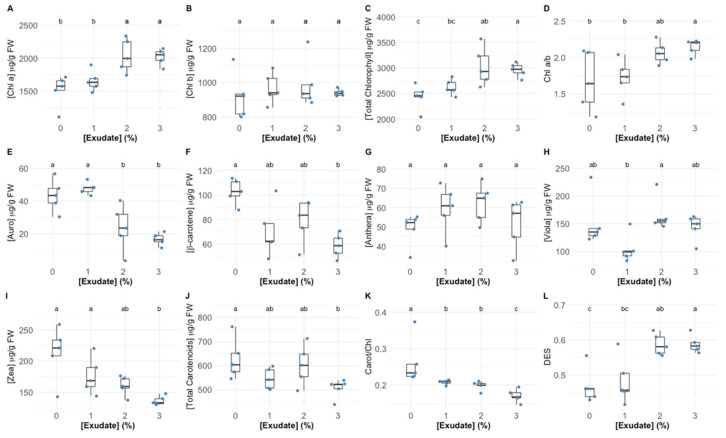
*Thellungiella halophila* pigment concentrations (Chl a—chlorophyll *a* (**A**); Chl b—chlorophyll *b* (**B**); Total chlorophyll (**C**); Auro—auroxanthin (**E**); β-carotene (**F**); Anthera—antheraxanthin (**G**); Viola—violaxanthin (**H**); Zea—zeaxanthin (**I**)), classes (total carotenoids (**J**)) and ratios (Chl a/b—chlorophyll *a* to chlorophyll *b* ratio (**D**); Carot/Chl—total carotenoid total to chlorophyll ratio (**K**); DES—De-Epoxidation State (**L**)) in leaves exposed to *Asparagopsis armata* exudate at the proposed target concentrations at the end of the trial period, (N = 5 per treatment; letters denote significant differences between treatments at *p* < 0.05).

The exposure to 1% exudate concentration did not alter the chlorophyll *a* leaf concentration (Figure 4A), while the higher tested concentrations led to a significant increase in this pigment concentration. Comparatively, the exudate application did not affect the leaf chlorophyll *b* concentration (Figure 4B). Considering the leaf total chlorophyll content (Figure 4C), it could be observed that the exudate application led to significant increases in this pigment pool, more evident in the plants exposed to the highest exudate concentration. If the ratio between these two main pigments is compared (Figure 4D), it can be seen that this parameter was not affected by exposure to the 1% exudate, whilst the application of 3% exudate concentrations led to a significantly higher chlorophyll *a* to *b* ratio (Chl a/b) values. Analysing the auroxanthin leaf concentration (Figure 4E), this was found to be significantly depleted upon the application of 2% and 3% exudate concentrations, while for β-carotene (Figure 4F), this depletion was more significant in the plants exposed to the highest exudate concentration. Regarding the pigments that are enrolled in the xanthophyll cycle, it was found that the exudate application did not change the concentration of antheraxanthin (Figure 4G), whilst violaxanthin suffered a significant reduction in the plants exposed to the 1% exudate concentration, in comparison with that observed for the plants exposed to the 2% exudate concentration (Figure 4H); the zeaxanthin content was severely depleted in the plants exposed to the highest *A. armata* exudate dose (Figure 4I). These changes led to a significant increase in the DES index in the plants exposed to the highest exudate concentrations (Figure 4L). All these changes in the carotenoid content are summarized in the significant depletion observed in the leaf total carotenoid content (Figure 4J). Comparing the total carotenoid and total chlorophyll contents in the leaves exposed to the different *A. armata* exudates it was found that the ratio between the concentration of these classes of pigments suffered a significant decrease in the plants exposed to the 1% and 2% exudate, this depletion being even more marked in the plants exposed to the highest exudate dose (Figure 4K).

### 3.3. Leaf Fatty Acid Profile

**Figure 5:**
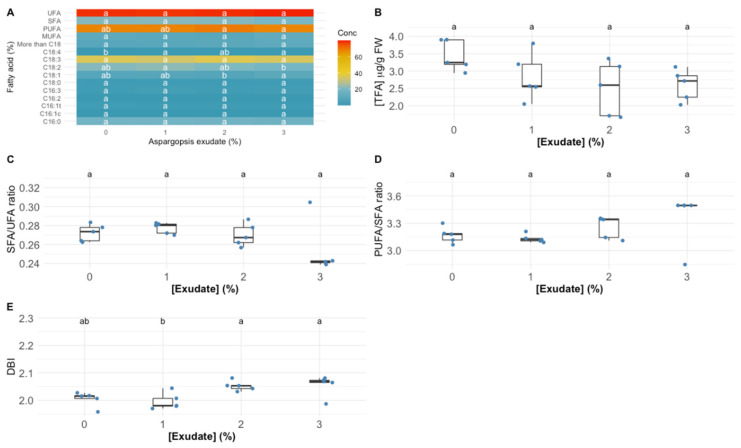
*Thellungiella halophila* leaf individual fatty acid and unsaturation classes (**A**) relative concentrations (UFA—unsaturated fatty acids; SFA—saturated fatty acids; PUFA—polyunsaturated fatty acids; MUFA—monounsaturated fatty acids) (%, average ± standard error, N = 5), total fatty acid content (TFA) (**B**), unsaturation ratios (**C**,**D**) and double-bound index (DBI) (**E**) in the plants exposed to *Asparagopsis armata* exudate at the proposed target concentrations at the end of the trial period (N = 5 per treatment; letters denote significant differences between treatments at *p* < 0.05).

In terms of individual fatty acid relative concentration, the exposure to the different tested exudate concentrations led to significant changes in oleic acid (C18:1), linoleic acid (C18:2) and stearidonic acid (C18:4) fatty acids (Figure 5A). More specifically, the exposure to the highest tested exudate concentration led to a significant increase in the C18:1 and C18:4 leaf relative concentration in the plants exposed to the highest exudate concentration tested, whilst for the concentration of C18:2, the inverse trend was observed, with a significant depletion of this fatty acid relative concentration in the plants exposed to the 3% *A. armata* exudate. Regarding the total fatty acid (TFA, Figure 5B), the exudate application did not change this parameter significantly in the leaves of the tested plants, despite a tendency for a decrease; this led to a significant decrease in the total PUFA concentration in the leaves of the plants exposed to the highest exudate concentration when compared to the individuals exposed to the 2% *A. armata* exudate. Regarding the DBI value, it was found the plants exposed to 2% and 3% showed a significantly higher value of these parameters when compared to the plants exposed to the 1% exudate (Figure 5E), but no significant changes were detected among the plants subjected to the different exudate concentrations in the SFA/UFA and PUFA/SFA ratios (Figure 5C,D, respectively).

### 3.4. Oxidative Stress

**Figure 6:**
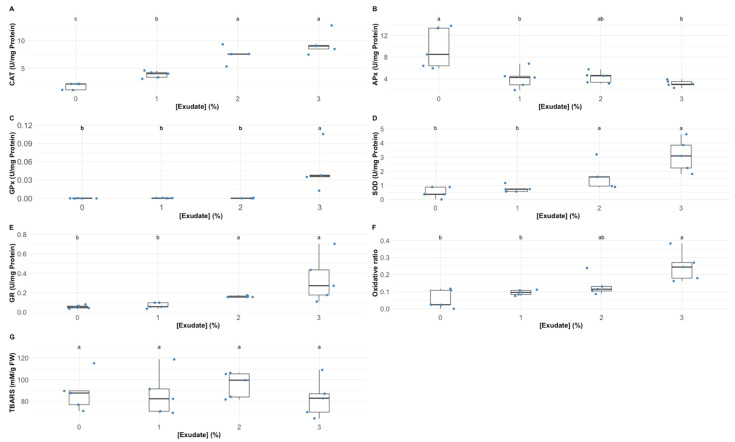
*Thellungiella halophila* leaf oxidative stress biomarkers (CAT—catalase activity (**A**); SOD—superoxide dismutase activity (**B**); GR—glutathione reductase activity (**C**); APx—ascorbate peroxidase activity (**D**); GPx—guaiacol peroxidase activity (**E**); oxidative ratio (**F**); and TBARS—thiobarbituric acid reactive substances (MDA equivalents) (**G**)) in the plants exposed to *Asparagopsis armata* exudate at the proposed target concentrations at the end of the trial period (N = 5 per treatment; letters denote significant differences between treatments at *p* < 0.05).

Analysing the oxidative stress enzymatic and non-enzymatic biomarkers, several differences could be detected in the plants exposed to the different exudate concentrations (Figure 6). Catalase (CAT), superoxide dismutase (SOD) and glutathione reductase (GR) leaf activities (Figure 6A–C, respectively) were significantly enhanced upon the application of the 2% and 3% *A. armata* exudate. On the other hand, ascorbate peroxidase (APx) showed significantly lower activity values in the plants exposed to 1% and 3% *A. armata* exudate (Figure 6D). Only the plants exposed to 3% exudate concentration showed a significant enhancement in guaiacol peroxidase (GPx) activity (Figure 6E). Regarding the balance between SOD and peroxidasic activity, here analysed throughout the oxidative ratio (Figure 6F), it was found that this parameter suffered a significant increase in the plants exposed to the 2% and 3% *A. armata* exudate (Figure 6F). Regarding the leaf TBARS concentration, the exudate application did not induce any significant changes (Figure 6G).

### 3.5. Biomarker Integrated Profiles

**Figure 7:**
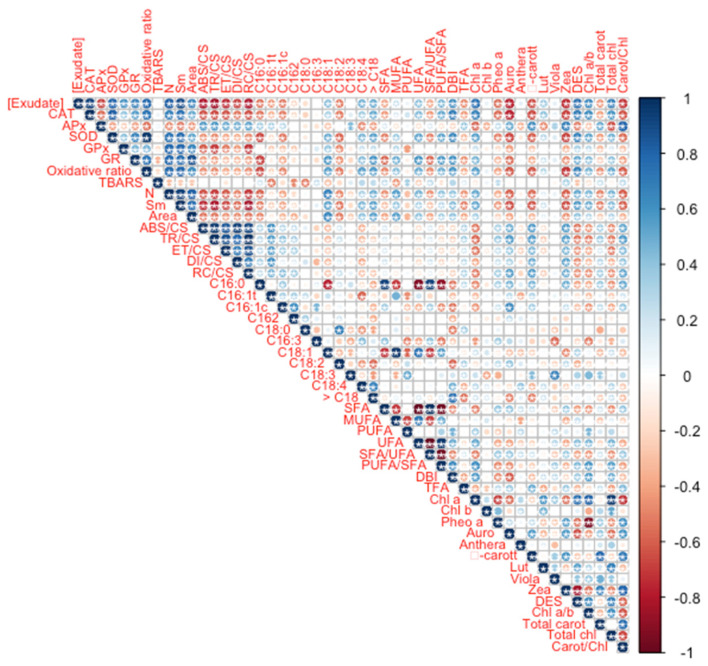
*Thellungiella halophila* leaf oxidative stress biomarkers, fatty acid and pigment profiles Spearman correlations with the *Asparagopsis armata* exudate at the proposed target concentrations at the end of the trial period (N = 5 per treatment; asterisks denote significant correlations at *p* < 0.05).

Analysing the oxidative stress biomarkers, it is possible to observe that, except for APx and TBARS, all showed a direct significant correlation with the *A. armata* exudate concentration applied (Figure 7). APx showed an inverse significant correlation with the exudate dose applied. Concerning the phenomological energy fluxes (ABS/CS, TR/CS, ET/CS, and DI/CS) as well as the oxidized reaction centre density (RC/CS), all showed an inverse and significant correlation with the exudate concentration to which the plants were exposed. Regarding the quinone pool functioning parameters (N, Sm and size of the oxidized quinone pool, here represented by the area above the Kautsky chlorophyll *a* induction curve), these were found to be positively and significantly correlated with the exudate concentration applied. Observing the dose-relationship between the fatty acid traits and the exudate concentration applied, the total leaf fatty acid content (TFA), SFA, SFA/MUFA and the relative amounts of C16:0C16:1 cis and trans, hexadecadienoic acid (C16:2) and C18:2 were inversely and significantly correlated with the concentration of exudate to which the plants were exposed. In opposition, MUFA, UFA, PUFA/SFA and DBI and the percentages of C18:1, C18:4 and the fatty acids with more than 18 carbons were found to be directly and significantly correlated with the *A. armata* exudate concentration applied. As for the leaf pigment profiles, chlorophyll *a*, lutein, DES, chlorophyll *a* to *b* ratio (Chl a/b) and total chlorophyll content was found to be significantly and directly correlated with the exudate concentration to which the plants were subjected. On the other hand, pheophytin a, auroxanthin, β-carotene, zeaxanthin, total leaf carotenoid content and the total carotenoid to total chlorophyll content ratio were found to be inversely and significantly correlated with the *A. armata* exudate concentration applied. Additionally, a high number of significant correlations were found between several of the analysed traits, highlighting the intrinsic relationship between the analysed biophysical and biochemical pathways. 

**Figure 8:**
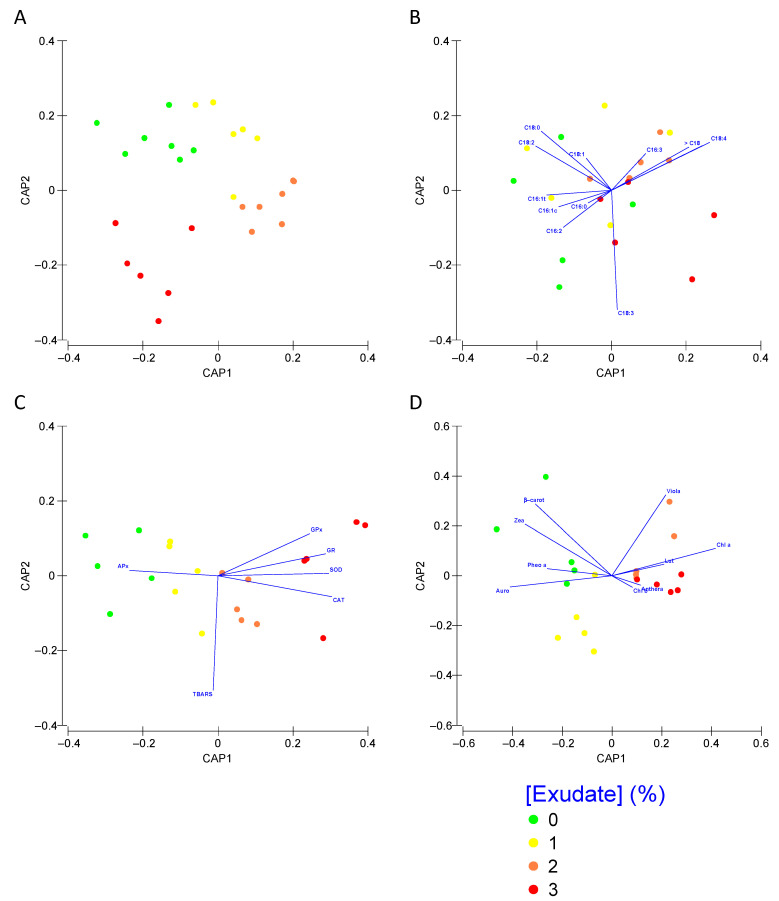
*Thellungiella halophila* photochemical (**A**), fatty acid (**B**), oxidative stress biomarkers (**C**) and pigment (**D**) profiles, assessed using multivariate Canonical Analysis of Principal Coordinates (CAP), of the plants exposed to the different *Asparagopsis armata* exudate concentrations tested at the end of the trial period (N = 5 per treatment).

Analysing each set of traits within a multivariate analysis approach, it is possible to depict which metabolic groups can be used as biomarker profiles of plant exposure to the *A. armata* exudate cocktail (Figure 8). Applying this approach to the Kautsky curve dataset (Figure 8A), it is possible to observe that this approach can efficiently (78.9% correct classification efficiency) distinguish the plants exposed to the different treatments. As absorbed in the univariate analysis, the fatty acid profile showed very little or no differences in the plants exposed to the different *A. armata* doses. Using a multivariate analysis with the whole fatty acid profile as input, it was found that these biomarkers have a very low resolution (40% correct classification efficiency) in depicting the plant samples exposed to the different *A. armata* exudate concentrations (Figure 8B). Regarding the oxidative stress biomarkers profile (Figure 8C), as a first approach, a separation is evident, observed from the first CAP axis, between the plant samples exposed to the 0% and 1% exudate and the samples exposed to the highest tested concentrations of this cocktail. Moreover, the classification efficiency of this multivariate approach gathers a global classification efficiency of 80% when considering the oxidative stress biomarkers as descriptors. A similar separation could be observed when using the pigment profile as descriptors of the plant exudate exposure, with a first and clear separation along the canonical first axis and an overall classification efficiency of 70% (Figure 8D).

## 4. Discussion

Marine macroalgae have been highly targeted for their potential as sources of promising biomolecules for a wide array of applications, including for agricultural purposes [8,9]. If these sources are provided from invasive species, their value is increased in terms of both potential biomolecule application and invasion control [10,17,40,41,42]. This is the case of the red algae *A. armata*, well known for its aggressiveness in the ecosystems that it invades and for its chemical arsenal to displace a wide number of species [12,13,14]. To test the biopesticide potential application of an *A. armata* exudate cocktail, a halophyte model weed (*T. halophila*) [21] was selected to avoid masking the effects of the saline cocktail carrier (seawater) in the photochemical and biochemical pathways here evaluated. 

Man-made pesticides are normally designed to target specific biochemical pathways, such as photosynthetic metabolism, to impair weed growth [43]. The first observable effects of the *A. armata* exudate cocktail application were the changes in intensity and shape of the OJIP transient induction curve with increasing concentrations of the applied exudate, leading to lower fluorescence intensities in all timesteps of the curve. This observation was previously detected in other works focusing on pesticide applications in plants and microalgae [4,44]. Although this is the first evidence that some impacts driven from exudate exposure are occurring in the photochemical processes of the tested plants, these can also be translated into more specific photochemical traits. As abovementioned, the functioning of the quinone pool, essential for electronic transport between photosystems, was severely affected upon the application of the *A. armata* cocktail. A high (longer) quinone redox turnover rate, along with a higher energy requirement to close all the reaction centres and an increasing value of the oxidized quinone pool size, indicate a lower availability of this pool for electron transport. This mode of action is very similar to the one observed upon the application of several major synthetic biocides, such as Basagran, Bromicide, Lumax and Gramoxone, where impairment of the electron transfer between the quinones Q_A_ and Q_B_ was observed [45,46,47]. This inevitably led to a reduction in the electron transport energy flux (ET/CS). Previous reports have shown that traditional pesticides application can lead to interruptions in the photosynthetic electron transport chain, leading to the concomitant inhibition of adenosine triphosphate (ATP) production and carbon fixation [47], and thus inhibiting biomass production, the main target of any herbicide application. Moreover, the plants exposed to the *A. armata* cocktail showed a significant dose-related reduction in the absorbed (ABS/CS) and trapped (TR/CS) energy flux, indicating a lower efficiency of the plants in harvesting light and generating the necessary excitons from the captured photons. This was also previously reported for the action of another group of pesticides (indole derivatives), where a decrease in all the phenomological energy transduction energy fluxes was also observed [48]. Glyphosate, a commonly used herbicide, induces the same mechanism [4]. A tentative counteractive measure towards this reduced energy harvest ability could also be observed in the present study. The plants exposed to the *A. armata* cocktail showed an increase in the light-harvesting centres (LHCs), here evaluated through the Chl a/b ratio. This was also previously reported upon the application of clomazone herbicide in sensitive varieties [49]. Nevertheless, our data indicate that this tentative counteractive measure did not produce any significant effects, with the absorbed and trapped energy fluxes remaining low, even in the plants with a high Chl a/b ratio. Additionally, and although with a decreasing trend, some energy dissipation is still observed, notwithstanding the fact that this was also found to be reduced upon the application of the macroalgae cocktail. The lack of efficient energy dissipation has several consequences at the photochemical level, such as potential photoinhibition processes [50], but also at the biochemical level, leading to the accumulation of deleterious and excessive redox potential [4,51]. To avoid an oxidative burst, the plant has mechanisms to quench the generated reactive oxygen species (ROS). At this level, all the evaluated antioxidant enzymatic activities, apart from APx, showed a significant dose-related increase upon the exudate application. Nevertheless, it is worth noticing the oxidative ratio value below 1, indicating a higher peroxidasic activity when compared with superoxide dismutase activity. In an equilibrium condition, the ratio tends to 1, indicating an equivalent rate between hydrogen peroxide generation from SOD superoxide dismutation and its metabolization by peroxidases. In this case, the peroxidasic activity is functioning at a much higher rate than SOD, indicating hydrogen peroxide generation from sources other than from SOD activity. Nevertheless, it should be noted that the remain signs of stress detected indicate that the peroxidasic activity was not sufficient to mitigate the oxidative damage caused by the exudate of *A. armata*. This is concomitant with the mode of action previously reported for paraquat-based herbicides [52]. This herbicide is also known as methyl viologen and can accept electrons directly from the FeS-clusters of PSI, generating a radical anion that can be re-oxidized by molecular oxygen, producing ROS directly, namely, in the form of H_2_O_2_ [52]. Regarding pigment-based energy dissipation mechanisms, plants are known to have two major pathways: through auroxanthin [53] or the xanthophyll de-epoxidation cycle [54]. Our data indicate that in the tested plants only the second mechanism was triggered. Increased de-epoxidation of the luminal violaxanthin pool (higher DES index) towards zeaxanthin decreases an overload of the photosynthetic light-harvesting apparatus, by dissipating the elevated reducing power accumulated within the stroma [54]. Nevertheless, for the maintenance of this process, carotenoids are diverted for xanthophyll production [54]. In the plants exposed to the *A. armata* cocktail, a significant depletion of carotenoid leaf content, namely, β-carotene, was observed. This carotenoid is not only the precursor of xanthophyll production, but it can act as a ROS quencher, due to its high antioxidant power [55]. It is reasonable to admit that this condition of depleted β-carotene in the plants exposed to *A. armata* exudate, as well as of the carotenoid pool, under prolonged exposure, can eventually lead to a breakdown in xanthophyll production, reducing energy dissipation and the antioxidant defences of the plant. This mechanism was also observed upon the application of the so-called Bleaching Herbicides, designed to disrupt carotenoid metabolism [56].

Fatty acids are major components of cellular membranes, including plastidial ones, and also key molecules in cellular trafficking and signalling [22]. In terms of leaf fatty acids, there was an inverse correlation between their total content and the concentration of exudate to which the plants were exposed, indicating membrane remodelling. Moreover, the increase in the double bond index results from an increase in the number of double bonds introduced in fatty acids by desaturases, reinforcing this membrane remodelling. Stress leading to over-accumulated ROS in plant cells can result in lipidic peroxidation, initiated by hydrogen abstraction or the addition of oxygen radical, with consequent damages of polyunsaturated fatty acids (PUFA) [57]. However, in our case, lipid peroxidation values (TBARS) remained constant, indicating that a protection mechanism is present. It has been suggested that unsaturated C18 fatty acids themselves can act as antioxidants; that is, they can directly react with and thus consume ROS [55], and the increase in their proportion in membranes, here observed, can represent a response mechanism to the applied stress. Moreover, it is interesting to notice that during the 7 days of exposure, a dose-related reduction in the levels of the chloroplastidial C16:1*t* (trans-hexadecenoic acid) was observed. This fatty acid is exclusively present in the chloroplast, being generated by a membrane-bound desaturase acting on C16:0, esterified to phosphatidylglycerol (PG), which is the only phospholipid present in thylakoids [58]. A reduction in this fatty acid has been linked to a reduction in the chloroplastidial electron transport chain stability and a lower photochemical performance [22,58,59,60]. These mechanisms are also reported for some herbicide formulations, namely, in the biocides based in pyridazinones, where a significant depletion of this fatty acid is observed along with a reduction of the plant physiological fitness [61].

Overall, the biomarker multivariate profiles reinforced the patterns discussed thus far. Plant photochemistry, pigments and oxidative stress biomarkers were highly affected by the exposure to the *A. armata* cocktail. These multivariate profile effects are more perceivable and also produce more reliable and efficient biomarkers. Non-invasive bio-optical tools that evaluate plant photochemistry preciously have been highlighted as among the most efficient tools to address plant stress [37,62,63]. In the present study, regardless of the high discrimination accuracy of the chlorophyll *a* fluorescence-derived biomarkers (78.9% correct classification accuracy), these were not the most efficient in depicting the *A. armata* exposure in the test plants. On the other hand, the oxidative stress biomarkers gathered the highest discrimination accuracy as biomarkers. In the past, it was already observed that these biomarkers can have a very good, dose-efficient response, with high performance under multivariate analysis in depicting the effects of a wide variety of toxic compounds in autotrophic organisms [38]. Regarding pigment composition, the results here attained are in line with previous work, where the pigment profiles can produce discrimination efficiencies with a medium-high classification accuracy [4]. Nevertheless, the evaluated biomarker traits constitute a highly robust dataset for the evaluation of the effects of an *A. armata* exudate cocktail on plants.

## 5. Conclusions

The cocktail of the exudate produced by the red seaweed *A. armata* induces a high degree of stress when applied in plants, sharing several effects and modes of action with several major synthetic biocides (such as basagran, bromicide, lumax, gramoxone, indole derivatives, clomazone, paraquat and pyridazinones), which impair several aspects mostly related to the plant energetic metabolism, with severe reductions in the chloroplastidial electron transport and consequent reduction of ATP generation and CO_2_ harvesting, eventually leading to plant death. This way, the *A. armata* exudate cocktail presents a high potential to be applied as a biopesticide, being a green and sustainable eco-friendly solution to reduce environmental contamination by hazardous chemical substances from human origin. In addition to the here-described biocide potential from a low-cost marine resource, an added value would be the resultant increased harvesting of this invasive seaweed, thus promoting more healthy and diverse shores. In comparison with the traditional chemical/artificial pesticides, the natural origin of this cocktail is also a matter of added-value in terms of increased public acceptance and of reduced environmental impact. The greatest opportunity is the fact that the extractive process leaves an intact and substantial by-product that may be further used for the current markets already using this seaweed, given that it does not lose the market target bioactivity. A study of the remaining biomass bioactivities (after exudate release) will be paramount to set the foundations for its use in an *A. armata* biorefinery, where this seaweed may serve several markets and thus present multiple revenue opportunities in a blue circular-economy framework.

## Figures and Tables

**Table 1:** Summary of the fluorometric analysis parameters and their description.

Variable	Definition
Area	Corresponds to the oxidized quinone pool size available for reduction and is a function of the area above the Kautsky plot.
N	Reaction centre turnover rate.
S_M_	Corresponds to the energy needed to close all reaction centres.
ABS/CS	Absorbed energy flux per cross-section.
TR/CS	Trapped energy flux per cross-section
ET/CS	Electron transport energy flux per cross-section.
DI/CS	Dissipated energy flux per cross-section.
RC/CS	The number of available reaction centres per cross-section.

## Data Availability

Not applicable.
